# An intestinal histiocytic sarcoma in a collared peccary (*Pecari tajacu*): a case report

**DOI:** 10.1186/s13028-025-00828-3

**Published:** 2025-09-02

**Authors:** Jael Soares Batista, Radan Elvis Matias de Oliveira, Wanderson Lucas Alves dos Santos, Ana Caroline Freitas Caetano de Sousa, Igor Renno Guimarães Lopes, João Augusto Rodrigues Alves Diniz, Thalita Evani Silva de Oliveira, Robério Gomes Olinda, Erick Platini Ferreira de Souto, Moacir Franco de Oliveira

**Affiliations:** 1 Department of Animal Sciences (DCA), Federal University of the Semi-Arid Region – UFERSA, Avenida Francisco Mota, 572, Costa e Silva, Mossoró, 59625-900 RN Brazil; 2 Verum Diagnostics, Animal Laboratory, Rua Zacarias Costa Camargo, 490, Remanso Campineiro, Hortolândia, 13184-280 SP Brazil; 3 Cearense Diagnostics Laboratory, Rua Doutor Walter Porto, 239, Parque Iracema, Fortaleza, 60822-250 CE Brazil

**Keywords:** Immunohistochemistry, Neoplasm, Tayassuidae, Wildlife pathology

## Abstract

**Background:**

Research on cancer in wild animals provides important insights into the mechanisms of carcinogenesis. Histiocytic sarcomas comprise a rare malignant macrophage-dendritic cell lineage neoplasm in wildlife. This study reports a case of histiocytic sarcoma in the small intestine of a collared peccary (*Pecari tajacu*), describing its clinical, anatomopathological, and immunohistochemical aspects.

**Case presentation:**

A six-year-old male collared peccary maintained in captivity at a facility in Northeastern Brazil presented progressive weight loss, diarrhea, anorexia, dyspnea, lethargy, abdominal distension, bristled fur, and pale mucous membranes. A complete blood count indicated a mild degree of anemia and moderate leukocytosis. Treatment included anti-inflammatories and antibiotics; however, on the 18th day after initial presentation, the animal was found dead in its enclosure. An anatomopathological examination revealed that the animal exhibited poor body condition, scant body fat with a gelatinous appearance, hydrothorax, pulmonary edema, and ascites. Thickening of the duodenal wall was observed, along with the presence of a yellowish-white tumor. Histopathological examination of the affected intestinal segment revealed a neoplastic proliferation of round cells with large, hyperchromatic nuclei, prominent nucleoli, and a high mitotic index (20 mitoses per high-power field). Numerous multinucleated and binucleated giant cells were present. The neoplastic cells extensively infiltrated all layers of the intestinal wall, from the mucosa to the serosa. Immunohistochemical analysis showed strong positivity for macrophage/mononuclear phagocytic lineage markers (CD18, IBA-1, and lysozyme), while negative for T-cell (CD3), B-cell (CD79), and plasma cell (MUM1) markers. The proliferation index assessed by Ki-67 was approximately 60%.

**Conclusions:**

The histopathological and immunohistochemical findings confirmed the diagnosis of intestinal histiocytic sarcoma in a collared peccary, representing the first documented case of this neoplasm in this species.

## Background

The collared peccary (*Pecari tajacu*), a member of the Tayassuidae family, inhabits a wide range of habitats, from humid tropical forests to semi-arid regions, and adapts well to diverse conditions. The survival of this species is facilitated by physiological and behavioral adaptations, allowing this animal to consume a variety of foodstuffs, such as fruits, leaves, roots, cacti, and tubers [1, 2]. Ecologically, collared peccaries are important seed dispersers, contributing to ecosystem equilibrium [3].

Captive breeding has allowed for the diagnosis of several diseases that affect collared peccaries, also providing guidance to commercial breeders [4]. The study of cancer in wild animals is important for elucidating unique mechanisms of carcinogenesis [5]. Several types have, in fact, been reported in captive collared peccaries, such as squamous cell mammary carcinomas [4, 6], extramedullary plasmacytomas [7], and splenic hemangiomas [4]. This demonstrates the importance of advancing scientific research to enhance the diagnosis of neoplastic diseases that may affect this species in captivity.

Histiocytic sarcomas (HS) comprise a malignant macrophage-dendritic cell lineage neoplasm [8] exhibiting variable biological behavior, although most malignant neoplasms often present a poor prognosis. Histiocytic disorders are less frequently reported in wild mammals, whereas they are well documented in dogs and cats [9, 10].

Immunohistochemistry is an important tool in distinguishing histiocytic tumors from other neoplasms with similar histological appearance. This allows for the establishment of definitive diagnoses and more accurate prognoses [9]. In this context, a case of HS in the small intestine of a collared peccary is reported herein, alongside clinical, anatomopathological, and immunohistochemical aspects.

## Case presentation

A six-year-old adult male collared peccary, weighing 23 kg, born in captivity at the Center for Multiplication of Wildlife Animals belonging to the Federal University of the Semi-Arid Region (CEMAS/UFERSA), located in Mossoró, Rio Grande do Norte, Brazil (5°12’49.4"S and 37°18’36.7"W Gr), was attended on February 15, 2024. This center is registered as a scientific breeding facility with the Brazilian Institute of Environment and Renewable Natural Resources (Registration no. 478912).

The animal presented progressive weight loss, diarrhea, anorexia, dyspnea, lethargy, abdominal distension, bristled fur, and pale mucous membranes. As a complementary test, only the complete blood count was performed, which indicated a mild degree of anemia (erythrocytes (x10^6^/µL): 6.0; hematocrit (%): 40.0; hemoglobin (g/dL): 16.0; MCV (fL): 50.0; MCHC (g/dL): 31.0) and moderate leukocytosis (leukocytes (x10^3^/µL): 19.50; neutrophils (x10^3^/mL): 13.95; lymphocytes (x10^3^/mL): 3.15; eosinophils (x10^3^/mL): 0.2; monocytes (x10^3^/mL): 2.20), compared to the normal reference values for the species [11]. Treatment was initiated with an anti-inflammatory steroid (dexamethasone 0.1 mg/kg) once a day for 7 days, alongside an antibiotic (enrofloxacin 10 mg/kg) once a day for 10 days, both administered intramuscularly. On the 18th day after initial presentation, the animal was found dead in its enclosure and was then sent to the Veterinary Pathology Laboratory, Department of Animal Sciences – UFERSA, where a necropsy was performed on the same day.

**Fig. 1:**
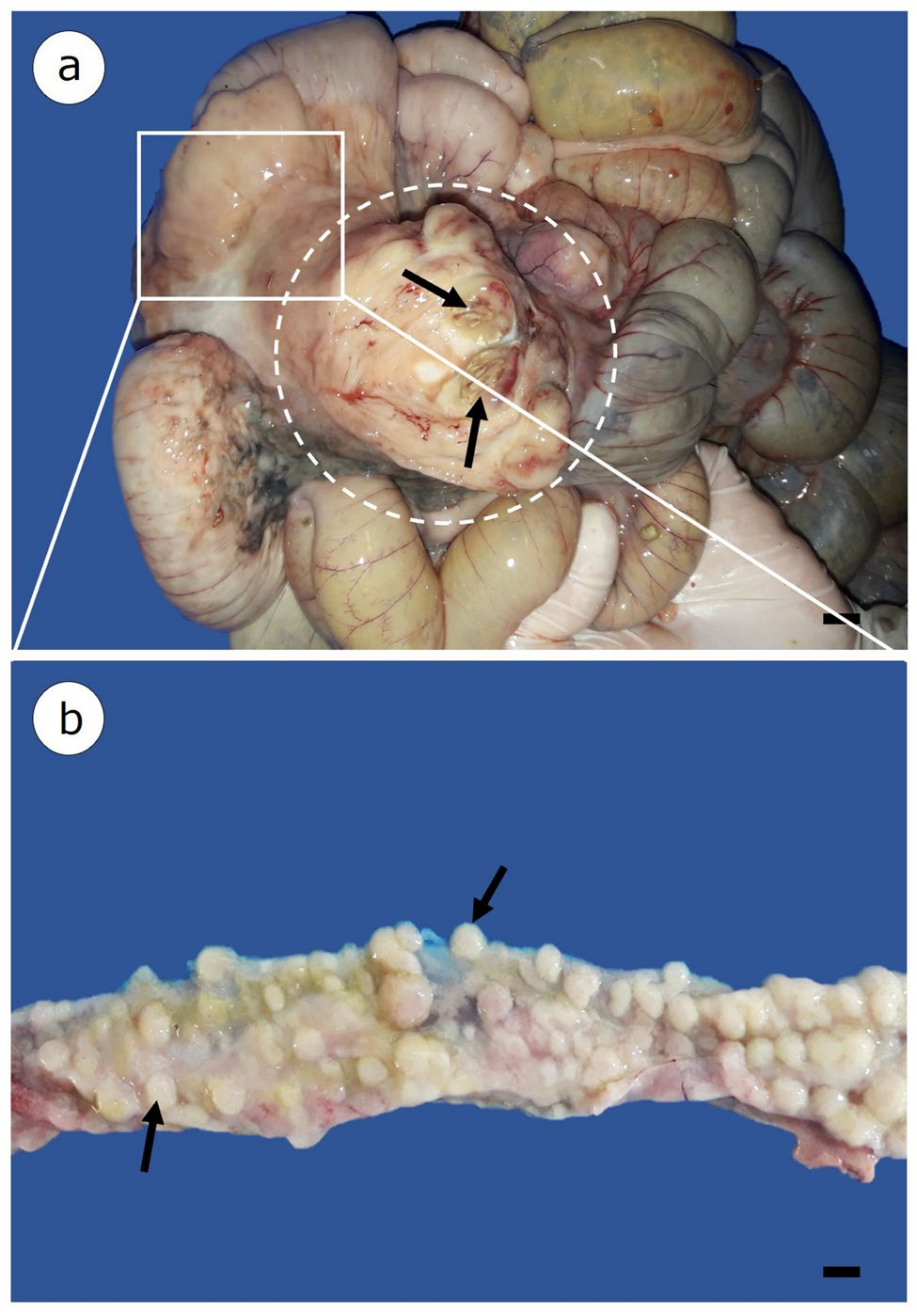
Macroscopic findings in the small intestine of a *Pecari tajacu* with histiocytic sarcoma. (**a**) Presence of a yellowish-white tumor (dashed circle) surrounded by a fibrous capsule, with ulcerated areas (arrows). Scale bar: 1.0 cm. (**b**) Presence of multiple nodular formations on the intestinal mucosa (arrows) in a segment collected near the tumor. Scale bar: 1 cm

The animal presented a poor body condition, scarce body fat with a gelatinous appearance, hydrothorax, pulmonary edema, and ascites. Upon necropsy examination, thickening of the small intestine wall was observed specifically in the duodenal segment, where a yellowish-white tumor measuring approximately 8.2 cm × 7.3 cm × 6.0 cm was also identified. The tumor was surrounded by a fibrous capsule and presented several ulcerated areas (Fig. 1a). A longitudinal section of the intestinal segment collected near the tumor indicated thickened walls and the presence of several yellowish-white nodular formations within the lumen (Fig. 1b). No tumors were observed in other organs.

Concerning the histopathological examination, tissue fragments from the brain, organs of the thoracic and abdominal cavities, skin, lymph nodes, and the tumor in the small intestine were fixed in a 10% formaldehyde solution buffered with phosphate (pH 7.3) and subjected to histological processing [12]. Section (5 μm) were obtained employing a LEICA RM 2165 microtome, adhered to glass slides, and maintained in a laboratory oven at 60 °C for up to 6 h for subsequent staining with hematoxylin and eosin. The slides were then observed under a Leica DM 500 HD light microscope coupled to a Leica ICC50W camera. Images were obtained using the LAS EZ Ink software. The mitotic count realized in 10 fields at 40x magnification / FN 22 / area of 2.37 mm² [13].

**Fig. 2:**
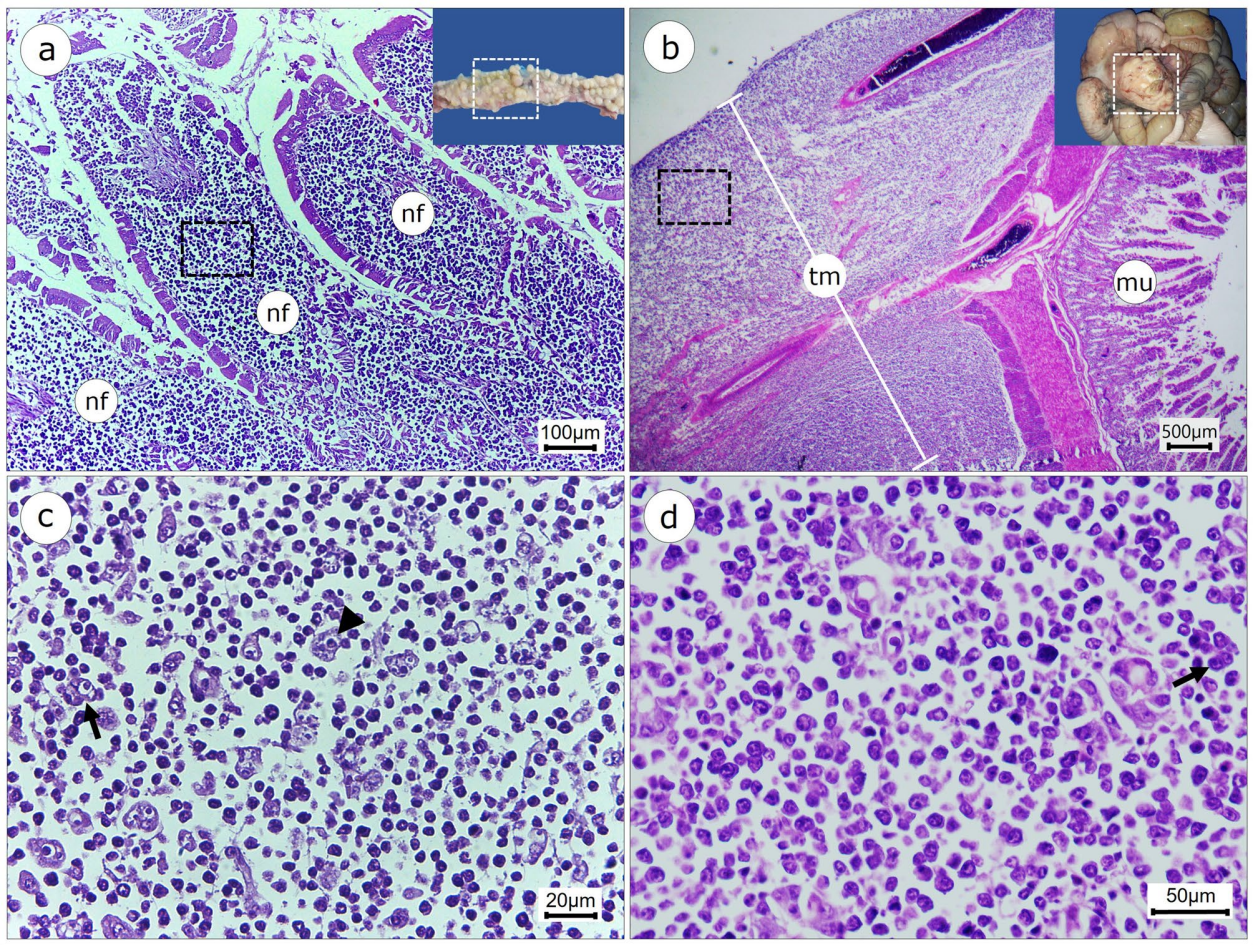
Histopathological findings in the small intestine of a *Pecari tajacu* with histiocytic sarcoma. (**a, b**) Transmural infiltration of neoplastic cells, from the mucosa to the serosa in the duodenum and the tumor, respectively. (Scale bars = 100 μm, 500 μm). (**c, d**) Inset of Fig. 2a and b, respectively, indicating the proliferation of giant, binucleated round cells (arrows) and multinucleated cells (arrowhead). (Scale bars = 20 μm, 50 μm). Legend: nodular formations in the mucosa (nf); area of the tumor in the serosal layer (tm); and mucosa (mu). Stains (**a, b, c, d**): hematoxylin and eosin

Histopathological analysis of both the tumor and the adjacent small intestine revealed the presence of round and polygonal neoplastic cells with giant, hypercolored nuclei, prominent nucleoli, marked pleomorphism, vacuolated cytoplasm and a high mitotic count (20 figures). The presence of giant, binucleated and multinucleated cells was also observed (Fig. 2c and d). The neoplastic cells infiltrated all histological layers, from the mucosa to the serosa. The remaining organs examined showed no significant histopathological alterations, and no metastases were observed.

Selected 4 μm tissue sections of the small intestine, fixed in formalin and embedded in paraffin, were used in the immunohistochemistry (IHC) protocol [14]. IHC assays were performed using the following monoclonal antibodies: CD3, CD79, MUM1, CD18, IBA-1, lysozyme, and Ki-67. CD3 and CD79 were used to identify T and B lymphocytes, respectively; MUM1 was applied for the detection of plasma cells; CD18, was used to assess the immunoreactivity of neoplastic cells of leukocyte origin; IBA-1 and lysozyme both for cells of the mononuclear phagocytic lineage, and Ki-67 was employed to evaluate cell proliferation.

Antigen retrieval was performed using citrate buffer (pH 6.0) and EDTA buffer (pH 8.9) in a pressure cooker system (Electrolux Pressure Cooker PCC10, São Paulo, SP, BR) for 3 min. Endogenous peroxidase activity was blocked with methanol and 3% hydrogen peroxide for 30 min in a dark chamber. The primary and secondary antibodies, along with their respective dilutions and controls, are listed in Table 1. Primary antibody incubation was carried out at 4 °C for 18 h, followed by incubation with the respective secondary antibody in a humid chamber for 30 min at 25 °C. Subsequently, the chromogen 3,3′-diaminobenzidine (DAB, Invitrogen, Camarillo/CA, USA) was applied for 3 min. The slides were counterstained with Harris’ hematoxylin, dehydrated through graded alcohol baths, cleared in xylene, and mounted with Entellan (Merck, Darmstadt, HE, DEU) and coverslips. Positive and negative controls were included in all IHC assays, with negative controls consisting of the replacement of the primary antibody with the corresponding diluent.

**Fig. 3:**
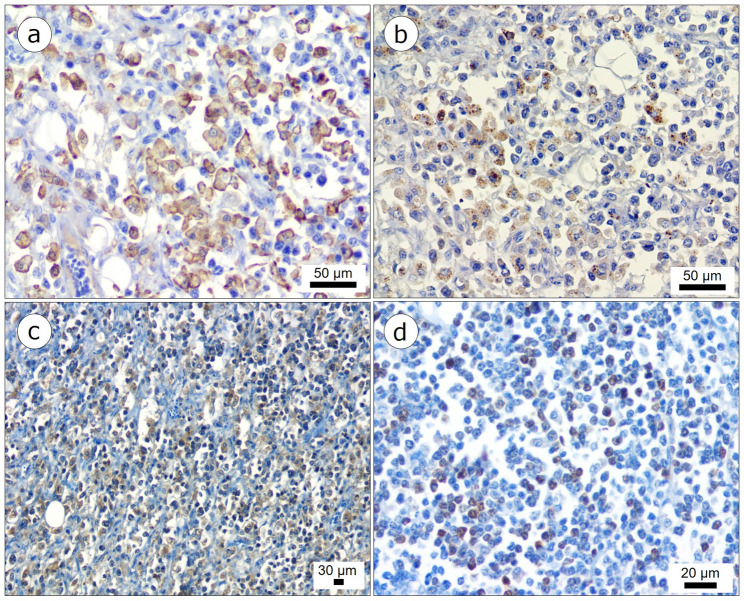
Immunohistochemical findings observed in the small intestine of a *Pecari tajacu* with histiocytic sarcoma. The neoplastic cells showed strong immunohistochemical positivity for IBA-1 (**a**), lysozyme (**b**), and CD18 (**c**). (**d**) The proliferative index showed strong Ki-67 positivity in approximately 60% of neoplastic cells. Stains (**a, b, c, d**): 3,3′-Diaminobenzidine (DAB) counterstained with hematoxylin. Scale bars: (**a, b**) = 50 μm; (**c**) = 30 μm; (**d**) = 20 μm

Immunohistochemical analysis revealed strong cytoplasmic positivity for IBA-1 in approximately 80% of the neoplastic histiocytes (Fig. 3a). Lysozyme was positive in around 50% of the samples (Fig. 3b), exhibiting moderate cytoplasmic immunolabeling. CD18 showed moderate immunoreactivity in approximately 55% of the cells (Fig. 3c), with evident cytoplasmic staining. Immunolabeling for CD3, MUM1, and CD79 was negative. The proliferative index, assessed by Ki-67, revealed strong intranuclear positivity in about 60% of the neoplastic cells (Fig. 3d). These findings support the diagnosis of intestinal HS in a collared peccary.

**Table 1:** Antibodies used in the immunohistochemical assays, including their dilutions, antigen retrieval methods, and source manufacturers

Primary Antibody	Clone	Dilution	Manufacturer	Antigen retrieval	pH	Secondary Antibody	Positive tissue control
CD3	F7.2.38	1:100	Santa Cruz Biotechnology (Dallas, TX, USA)	Citrate	5.6	Ervision Flex / HRP	Swine tonsil
CD79	JCB117	1:100	Sigma-Aldrich (St. Louis, MO, USA)	EDTA	8.9	Dako Agilent	Swine tonsil
MUM1	EP190	1:100	Bio SB (Santa Barbara, CA, USA)	EDTA	8.9	Dako Agilent	Dog tonsil
Ki67	MIB-1	1:400	Dako (Carpinteria/CA, USA)	EDTA	8.9	Dako Agilent	Swine small intestine
CD18	Monoclonal	1:80	Abcam (Cambridge, Cambs, UK)	EDTA	8.9	Dako Agilent	Swine tonsil
IBA-1	Polyclonal	1:450	Wako Chemicals (Richmond Richmond, VA, USA)	EDTA	8.9	Dako Agilent	Rat brain
Lysozyme	Polyclonal	1:300	Dako (Carpinteria, CA, USA)	Citrate	5.6	Dako Agilent	Dog myeloid leucemia

## Discussion and conclusions

Histiocytic diseases are rare in wild animals, but relatively common in dogs and cats [9, 15, 16]. Recently, HS has been diagnosed in four-toed hedgehogs (*Atelerix albiventris*) [17], Asian palm civets (*Paradoxurus hermaphroditus*) [18], Bengal tiger (*Panthera tigris tigris*) [19], Florida manatees (*Trichechus manatus latirostris*) [20], jaguars (*Panthera onca*) [21], and captive hybrid orangutan (*Pongo* sp.) [22]. To the best of our knowledge, this is the first HS report on a collared peccary.

Similar histopathological findings as those reported herein were observed in the literature. Histologically, HS is characterized by sheets of large, round to polygonal, discohesive cells exhibiting epithelioid to pleomorphic morphology, pronounced anisocytosis and anisokaryosis, and frequent binucleated and multinucleated cells. The cytoplasm is abundant, eosinophilic to vacuolated or foamy, and the nuclei are oval to irregular in shape, often with prominent nucleoli. High mitotic activity, evident tumor necrosis, and a prominent mixed inflammatory infiltrate in the background are also observed [15, 23, 24]. However, immunophenotypic confirmation is essential to differentiate HS from other mesenchymal tumors, particularly round cell tumors. The differential diagnosis is broad, with certain types of lymphoma being one of the primary considerations, given the morphological similarities between these neoplasms [25].

The diagnosis of HS was confirmed based on histopathological features, positive immunoreactivity for CD18, IBA-1, and lysozyme, and the exclusion of T cells, B cells, and plasma cell lineages through negative expression of CD3, CD79, and MUM1, respectively. Although other histiocytic markers such as CD68, CD163, and CD4 are available for the diagnosis of HS [23, 24], the immunohistochemical panel used in this study was considered adequate to rule out the main lymphoid differential diagnoses.

The infiltration of neoplastic cells observed in the intestine, primarily involving the mucosa to the serosa, compromises the structural and functional integrity of the intestinal wall. The damage to the mucosa, the main site of nutrient absorption, leads to significant malabsorption, which may result in profound nutritional deficiencies [26]. Therefore, as the tumor progressed, it resulted in the inability to properly absorb nutrients, electrolytes, and water, which may have led to systemic complications such as severe weight loss, hypoproteinemia, electrolyte imbalances, and dehydration. These complications were possibly determinants in the animal’s death.

Histiocytic sarcomas exhibit high metastatic rates [27]. In most cases, when metastasis occurs, symptoms vary depending on the affected location. Nonspecific symptoms such as progressive weight loss, diarrhea, anorexia, dyspnea, and lethargy are commonly reported [9, 10, 15, 16, 21, 22]. Although the affected collared peccary did not present metastasis, these clinical symptoms were also observed in the present study. The literature often reports a poor prognosis for HS, as this neoplasm exhibits aggressive progression and a rapidly progressive clinical course, resulting in short survival periods, particularly in cases of disseminated disease [28].

The high mitotic count and elevated proliferative Ki-67 (60%) index verified herein confirm that the collared peccary neoplasm was highly malignant, for similar cases in the literature [29].

This is the first report on HS occurrence in collared peccaries. Given the rarity of this neoplasm in the species, this report highlights the importance of conducting pathological and histopathological examinations. When combined with other methods, such as immunohistochemistry, these examinations allow for accurate diagnoses and assessments of disease progression, offering valuable insights into conditions affecting collared peccaries.

## Data Availability

The datasets used and/or analysed during the current study are available from the corresponding author on reasonable request.

## Declarations

### Prior publication

Data have not been published previously.

## Abbreviations

CD18: Integrin beta-2
CD3: Cluster of differentiation 3
CD79: Cluster of differentiation 79
IHC: Immunohistochemical analysis
MUM1: Multiple myeloma 1
IBA-1: Ionized calcium-binding adapter molecule 1
Ki-67: Cell proliferation marker
HS: Histiocytic sarcoma
